# Letter from Dr. John Seale in Reply to Brown *et al.*

**Published:** 1988-02

**Authors:** John R. Seale

**Affiliations:** 78 Harley Street London W1


**Letter from Dr. John Seale in Reply to Brown *et al.***

Brown *et al.* wisely state that, "misinformation will not . . . control . . . the Aids epidemic," but rashly assert, "infection has not been documented in Western families outside sexual contact or . . . traumatic blood contamination."

Centers for Disease Control (CDC) in Atlanta documented (1) that an American mother was infected by her one-year-old son; our Communicable Diseases Surveillance Centre documented (2) that an English woman was infected caring for her sick neighbour in the tradition of the good Samaritan; virologists documented (3, 4) in the Lancet that a little three-year-old German boy infected his six-year-old brother at play, and a physically impotent American sextagenarian infected his elderly wife. In addition CDC have documented (5) that three American nurses were infected following a single episode of minor, non-traumatic contamination of their skin with blood. In none of these documented cases was there any sexual contact, nor trauma to the person who became infected.

There is nothing particularly mysterious about these cases in the light of the known pathogenesis of HIV infection and elementary biological principles. Brown et al correctly state that HIV persists in the blood of infected people for life, but omit the facts that up to 25,000 infectious virions per millilitre have been detected in *serum* (6), yet none has been detected in *semen* (7, 8).

If an HIV virion reaches a macrophage of the next host transfer of infection can occur. Macrophages are present in large numbers within the superficial layers of the epidermis and mucosal surfaces. Consequently a little serum coming into contact with a microscopic abrasion or other epithelial discontinuity is sufficient to transmit: "blood to blood transfer" is not necessary, although particularly efficient. Obviously "appostion of eroded or raw inflamed areas . . . will give the virus an increased chance of transmission"; but this applies to areas on the lips, hands or any other part of the anatomy—not just the genitalia.

The pattern of disinformation about HIV transmission as exemplified by the letter from Brown et al is depressingly familiar. At first it is stated that a particular mode of transmission is theoretically impossible; such as child to child, patient to nurse, child to mother, or surgeon to patient. Then it is asserted that no case has been reported anywhere in the world—but when cases are reported they are selectively ignored. Finally, publication in journals of medical science of logical conclusions which can be drawn from known facts is usually suppressed, either in the editor's office, or by the peer review system of "expert referees". These "experts" in the case of Aids, are largely composed of medical members of a high risk group, determined to block HIV testing being carried out routinely on everybody irrespective of sexual peculiarities.

In those rare instances where an editor has the courage to publish facts and conclusions unacceptable to those whose aberrant sexual behaviour has been the major amplifier of the spread of HIV in our country, the editor is abused and the scientific integrity of the author is rubbished.

John R. Seale
78 Harley Street
London W1

**REFERENCES**

1. Centers for Disease Control (1986) Apparent transmission of HTLV-111/LAV from child to mother providing health care. *JAMA*; **255**, 1005–110.
2. GRINT, P. McEVOY, M. (1985) Two associated cases of AIDS. *Communicable Disease Report*. **42**, 4.
3. WAHN, W. KRAMER, H. H. VOIT, T. *et al* (1986) Horizontal transmission of HIV infection between two siblings. *Lancet* **2**, 694.
4. SALAHUDDIN, S. Z. GROOPMAN, J. E. MARKHAM, P. D. *et al* (1984) HTLV-111 in symptom-free seronegative persons. *Lancet* **2**, 1418–20.
5. Centers for Disease Control (1987) Human immunodeficiency virus infections in health-care workers exposed to blood of infected patients. *JAMA* **257**, 3032–39.
6. LEVY, J. A. KAMINSKY, L. S. MORROW, W. J. W. *et al* (1985) Infection by the retrovirus associated with acquired immune deficiency syndrome. *Ann.Intern.Med.* **103**, 694–9.
7. ZAGURY, D. BERNARD, J. LEIBOWITCH, J. *et al* (1984) HTLV-111 in cells cultured from semen of two patients with AIDS. *Science* **226**, 449–51.
8. HO, D. D. SCHOOLEY, R. T. ROSS, T. R. *et al* (1984) HTLV-111 in the semen and blood of a healthy homosexual man. *Science* **226**, 451–3.

